# Descriptive Histological and Histomorphometrical Comparison of Four Xenogeneic and Synthetic Bone Blocks for Mandibular Onlay Augmentation in Rabbits

**DOI:** 10.3390/jfb17090472

**Published:** 2026-09-17

**Authors:** Souichiro Honda, Daniele Botticelli, Erick Ricardo Silva, Samuel Porfirio Xavier, Giovanna Iezzi, Hitoshi Seo, Shunsuke Baba

**Affiliations:** 1 Department of Oral Implantology, School of Dentistry, Osaka Dental University, Osaka-573-1121, Japan; hondasoul25@gmail.com (S.H.); seo-h@cc.osaka-dent.ac.jp (H.S.); baba-s@cc.osaka-dent.ac.jp (S.B.); 2 ARDEC Academy, 47923 Rimini, Italy; 3 Department of Oral and Maxillofacial Surgery and Periodontics, Faculty of Dentistry of Ribeirão Preto, University of São Paulo, Ribeirão Preto 14040-904, Brazil; erick.silva@usp.br (E.R.S.); spx@forp.usp.br (S.P.X.); 4 Department of Medical, Oral and Biotechnological Sciences, University G. d’Annunzio of Chieti, 66100 Chieti, Italy; gio.iezzi@unich.it

**Keywords:** animal study, bone regeneration, onlay bone augmentation, block bone substitutes, interpenetrating bone network, synthetic biomaterials, xenogeneic bone substitutes

## Abstract

Background: This exploratory study provided a primarily descriptive comparison of two xenogeneic and two synthetic blocks for mandibular onlay augmentation in rabbits. Methods: Twelve rabbits received bilateral block grafts, providing 24 sites allocated to Bio-Oss^®^ Block, SP-Block, ReproBone^®^ Block, or Alos Block (*n* = 6/material). After 10 weeks, undecalcified sections were evaluated within standardized inferior and superior regions. Newly formed bone was the primary outcome; secondary outcomes included residual graft, IBN-like tissue, soft-tissue components, and cross-sectional augmented area. Mixed-effects models accounted for clustering within animals. Results: No statistically significant difference in newly formed bone percentage was detected among biomaterials (*p* = 0.475). Residual graft differed significantly (*p* < 0.001) and was lower for all test materials than for Bio-Oss^®^ Block. ReproBone^®^ Block consistently exhibited IBN-like tissue (17.3 ± 2.9%). SP-Block showed more intra-compartment soft tissue than Bio-Oss^®^ Block. Alos Block had the smallest augmented area (12.9 ± 6.1 mm^2^; adjusted *p* = 0.001 versus Bio-Oss^®^ Block), and extra-compartment soft tissue was present in five of six sites. Conclusions: Although newly formed bone percentages did not differ significantly, the biomaterials displayed distinct profiles of scaffold persistence, incorporation, tissue organization, and cross-sectional augmented area at 10 weeks. These features should be considered together when evaluating block biomaterials for onlay augmentation.

## 1. Introduction

Reconstruction of deficient alveolar ridges remains a major challenge in implant dentistry. Following tooth extraction, periodontal disease, trauma, tumor resection, or congenital defects, alveolar bone loss may compromise implant placement by reducing the available bone volume and altering the three-dimensional architecture of the residual ridge [1,2,3,4,5,6]. In these situations, bone augmentation procedures are frequently required to restore sufficient hard tissue volume and to allow prosthetically driven implant rehabilitation [7,8,9,10,11,12].

Autogenous bone has traditionally been considered the reference material for alveolar ridge augmentation because of its osteogenic, osteoinductive, and osteoconductive properties [4,5,7,8]. However, autogenous grafting is associated with relevant disadvantages, including donor-site morbidity, postoperative discomfort, increased surgical time, limited availability of graft volume, and possible graft resorption during healing [4,5,12,13,14]. These limitations have stimulated the development and evaluation of alternative biomaterials, including allogeneic, xenogeneic, and synthetic bone substitutes, with the aim of reducing morbidity while maintaining adequate regenerative potential and dimensional stability [12,14,15,16,17].

Block grafting has particular relevance in horizontal and vertical ridge augmentation because the block provides a stable three-dimensional scaffold that may contribute to space maintenance and reconstruction of the augmented contour [18,19,20]. Nevertheless, graft incorporation is strongly influenced by the biological configuration of the recipient site, graft stability, vascular supply, adaptation to the recipient bed, and the osteoconductive properties of the material used [1,21,22,23,24]. Previous experimental studies in rabbit mandibles comparing inlay and onlay block grafting showed that inlay grafts generally exhibit more favorable bone formation and incorporation than onlay grafts [23,24]. This advantage has been attributed to the self-contained morphology of the inlay defect, which provides increased contact with the parent bone and multiple sources for vascular and cellular ingrowth [23,24]. In contrast, onlay grafts are placed on a one-wall recipient bed and are therefore more dependent on graft stability, revascularization from the underlying bone, protection from soft tissue invasion, and the intrinsic osteoconductive and remodeling properties of the biomaterial [1,21,23,24].

In this context, the onlay model represents a biologically demanding condition for evaluating bone substitutes [1,21,23,24]. The recipient site offers limited bony contact, and bone formation must progress from the underlying cortical surface toward the external regions of the graft [23,24]. Previous experimental studies reported that deproteinized bovine bone mineral blocks used as onlay or inlay grafts may show limited incorporation, with newly formed bone mainly restricted to the basal region close to the recipient bone and, in some cases, connective tissue interposition between the graft and the parent bone [1,21]. Conversely, collagenic cancellous equine bone blocks have shown more favorable osteoconductive behavior, with newly formed bone growing along the trabecular structure of the graft and, in some specimens, reaching more peripheral regions [23,24].

The biological performance of bone grafting materials is influenced by their origin, composition, porosity, microarchitecture, degradation or resorption behavior, and space-maintaining capacity [25,26,27]. Deproteinized bovine bone mineral is commonly used as a reference xenogeneic material because of its well-documented osteoconductive properties and slow resorption profile, which may contribute to maintenance of the augmented volume but also result in substantial long-term persistence of residual biomaterial [28,29,30,31].

Collagenic cancellous equine bone blocks, in which the native collagen component is preserved during processing, have been developed to provide an osteoconductive scaffold with the potential for progressive remodeling [23,24,32]. Synthetic substitutes, including hydroxyapatite, β-tricalcium phosphate, biphasic calcium phosphates, and polymer–calcium phosphate composites, offer alternatives to animal-derived grafts and may exhibit markedly different patterns of scaffold persistence, degradation, bone ingrowth, and biological replacement [33,34,35,36,37]. Their performance should therefore not be assessed solely according to the percentage of newly formed bone, but also by considering residual scaffold, remodeling behavior, mode of graft incorporation, spatial distribution of bone formation, and dimensional maintenance of the augmented region.

However, comparative information on the histological behavior of block biomaterials of different origins and compositions when used as onlay grafts on the lateral aspect of the mandible remains limited. Because the onlay configuration represents a less favorable biological environment than contained or interpositional defects, this model may help reveal differences in osteoconductive potential, graft persistence, remodeling pattern, and bone formation among scaffolds with different structures and degradation characteristics [1,21,23,24]. The comparison of deproteinized bovine bone mineral, collagenic cancellous equine bone with preserved native collagen, and synthetic hydroxyapatite-based substitutes may therefore provide relevant information on histological healing patterns associated with scaffolds differing in reported composition, structure, and degradation characteristics in a non-contained augmentation model [1,16,17,21,23,24].

Accordingly, four commercially available biomaterials were selected to represent contrasting block-graft concepts: a widely used deproteinized bovine bone mineral with a reported slow-resorption profile, serving as the reference material; a collagen-containing equine xenograft; a biphasic calcium phosphate ceramic; and a polymer–hydroxyapatite composite. The aim of the present experimental study was to determine whether these materials, when standardized to comparable external dimensions and evaluated under identical onlay conditions on the lateral aspect of the rabbit mandible, would exhibit different histological healing and remodeling patterns after 10 weeks. The histological and histomorphometric analyses focused on newly formed bone and its spatial distribution, residual biomaterial, soft-tissue components, graft incorporation, and maintenance of the augmented cross-sectional area.

## 2. Materials and Methods

### 2.1. Ethical Considerations

The experimental protocol was approved by the Animal Ethics Committee of the Faculty of Dentistry of Ribeirão Preto, University of São Paulo, Brazil, on 12 June 2024, under protocol number 0115/2024. All experimental procedures were performed in accordance with the Brazilian regulations for animal experimentation and followed the principles of the ARRIVE 2.0 guidelines.

### 2.2. Study Design and Experimental Unit

This was a prospective, randomized, exploratory preclinical study in rabbits. Four different block biomaterials were evaluated as onlay grafts on the lateral aspect of the mandible after 10 weeks of healing.

A total of 12 rabbits were included. Each animal received two blocks of biomaterials, one on each hemimandible, according to a randomized allocation sequence. Therefore, each biomaterial was represented by six experimental sites. The lateral surface of the mandibular angle was used bilaterally as the recipient site. In all sites, the cortical bone was perforated to promote vascular and cellular supply from the underlying medullary compartment. The assigned block biomaterial was fixed in an onlay position using a titanium fixation screw and was covered with a collagen membrane.

The four experimental groups were as follows:

- Bio-Oss^®^ Block (Geistlich Pharma AG, Wolhusen, Switzerland);
- OsteoBiol^®^ SP-Block (Tecnoss^®^, Giaveno, Italy);
- ReproBone^®^ Synthetic Bone Graft (Ceramisys Ltd., Sheffield, UK);
- Alos Block (Allmed S.r.l., Lissone, Italy).

The primary outcome was the percentage of newly formed bone within the standardized regions of interest. Secondary outcomes included the percentages of residual biomaterial, interpenetrating bone network-like (IBN-like) tissue, intra- and extra-compartment soft tissues, and other components, as well as the cross-sectional area of the augmented region.

### 2.3. Experimental Animals and Eligibility Criteria

Twelve adult female New Zealand White rabbits, 5–6 months of age and weighing approximately 3.5–4.0 kg, were included in the experiment. The animals were housed individually at the Animal Facility of the Faculty of Dentistry of Ribeirão Preto, University of São Paulo, under controlled environmental conditions, with free access to water and a standard laboratory diet.

### 2.4. Acclimatization, Housing, Husbandry, and Environmental Enrichment

Following arrival at the University of São Paulo, the rabbits were acclimatized to the institutional animal facility for at least 7 days before surgery.

Throughout the study, all animals were housed individually in the same animal room under identical environmental conditions. Stainless-steel cages providing a surface area of 4500 cm^2^ per animal were maintained under a 12 h light/dark cycle, controlled temperature (20–22 °C), and 15–25 air changes per hour. Standard laboratory chow and filtered water were available ad libitum.

Environmental enrichment consisted of a plastic resting platform provided in each cage throughout the experimental period.

### 2.5. Sample Size Calculation

The sample size estimation was based on the percentage of newly formed bone, selected as the primary histomorphometric outcome. No previous data were available for a direct comparison of the four biomaterials evaluated as standardized onlay blocks on the lateral aspect of the rabbit mandible. Therefore, the expected variability was derived from a previous experimental study using a comparable rabbit mandibular block-graft model, in which the maximum standard deviation for newly formed bone was 7.4 percentage points [24].

Because the principal planned comparisons evaluated each test biomaterial against Bio-Oss^®^ Block, the sample size was approximated using a two-sided comparison between two independent group means. A difference of 15 percentage points was considered biologically relevant for this exploratory model, corresponding to a standardized effect size of 2.03. With a two-sided α level of 0.05 and a power of 80%, approximately five experimental sites per group were estimated to be required. Six sites per biomaterial were ultimately included to provide a modest allowance for uncertainty, variability, and possible specimen loss. The calculation was reproduced using the power.t.test function in R software, version 4.6.1 (R Foundation for Statistical Computing, Vienna, Austria).

Each rabbit contributed two hemimandibular experimental sites. Thus, 12 animals provided 24 sites, corresponding to six sites for each biomaterial. Allocation was performed at the site level and was not constrained within animals; consequently, some rabbits received the same biomaterial bilaterally. Because no reliable prior estimate of within-animal correlation was available for this experimental model, clustering of the two sites within each rabbit could not be incorporated into the sample-size calculation. The independent-groups calculation should therefore be regarded as an approximate planning estimate for detecting large material-related differences and may have overestimated the effective statistical power. The correlation between sites belonging to the same animal was subsequently accounted for in the analysis by including animal as a random intercept in the mixed-effects model.

### 2.6. Randomization, Allocation Concealment, and Blinding

Treatment allocation was generated electronically at the experimental-site level by an investigator not involved in animal selection, surgical procedures, or histological evaluation (S.P.X.). The allocation sequence was designed to obtain six sites for each biomaterial among the 24 available hemimandibular sites. No restriction was applied to force the placement of two different biomaterials within the same animal. Therefore, in some rabbits, the same biomaterial was assigned to both hemimandibles.

Allocation was concealed in opaque sealed envelopes, which were opened immediately before block placement. Histological specimens were coded before analysis. Complete masking of the examiner could not be guaranteed because of the different histological appearance of the tested biomaterials.

### 2.7. Biomaterials

**Figure 1:**
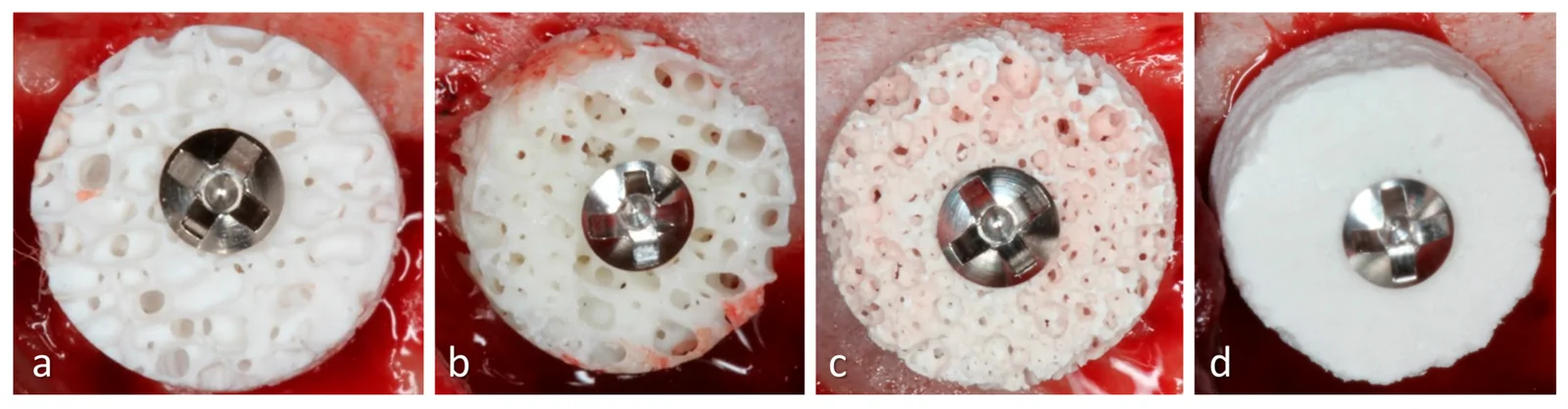
Clinical appearance of the four block biomaterials after shaping and fixation to the lateral aspect of the rabbit mandible with a titanium screw. (**a**) Bio-Oss^®^ Block; (**b**) OsteoBiol^®^ SP-Block; (**c**) ReproBone^®^ Block; (**d**) Alos Block. The blocks were standardized to approximately 7 mm in diameter and 3 mm in height before placement.

Four commercially available block biomaterials were evaluated. The materials were selected a priori to represent two xenogeneic and two synthetic block-graft concepts with contrasting biological origins, manufacturer-reported compositions, and anticipated remodeling profiles, with Bio-Oss^®^ Block serving as the reference material. Before implantation, each block was trimmed and shaped to obtain standardized cylindrical specimens measuring approximately 7 mm in diameter and 3 mm in height. Preparation was performed under copious sterile saline irrigation to limit overheating and to obtain comparable external dimensions among groups. A central hole was then prepared in each block to allow insertion of the titanium fixation screw. The following biomaterial blocks were used (Figure 1):

1. Bio-Oss^®^ Block (Geistlich Pharma AG, Wolhusen, Switzerland) was used as the deproteinized bovine bone mineral block. This xenogeneic biomaterial consists of a porous mineral matrix derived from bovine bone and was included as a slowly resorbing reference material.
2. OsteoBiol^®^ SP-Block (Tecnoss^®^, Giaveno, Italy) was used as the collagenic equine-derived block. This material consists of cancellous xenogeneic bone in which the bone matrix is not ceramized and the collagen component is preserved within the scaffold structure. In the present study, according to the manufacturer, the blocks were hydrated with sterile saline solution before fixation.
3. ReproBone^®^ Blocks (Ceramisys Ltd., Sheffield, UK) were used as the synthetic calcium phosphate block biomaterial. The material consists of a resorbable biphasic calcium phosphate ceramic composed of hydroxyapatite and β-tricalcium phosphate and is characterized by a highly interconnected porous structure.
4. Alos Block (Allmed S.r.l., Lissone, Italy) was used as the synthetic polymer–hydroxyapatite block. This material is described as a moldable, fully synthetic and resorbable block biomaterial, without components of biological origin. It consists of a polylactic/polyglycolic acid-based copolymer associated with porous non-sintered hydroxyapatite; the copolymer provides a temporary space-maintaining structure, whereas the hydroxyapatite phase acts as the mineral osteoconductive component.

After block fixation, all experimental sites were covered with an OsteoBiol^®^ Evolution Standard membrane (Tecnoss^®^, Giaveno, Italy). This resorbable, non-cross-linked collagen membrane originates from xenogeneic pericardium tissue and is characterized by a dense collagen-fiber matrix intended to protect and stabilize the grafted region [38]. Membrane permeability or cell occlusivity was not independently quantified in the present study. The membrane was trimmed with sterile scissors, hydrated when required, and adapted over the block and surrounding recipient bed.

The descriptions of the composition and structural characteristics of the four block graft materials were based on information provided by the manufacturers and the available literature. No independent physicochemical or microstructural characterization of the specific material batches was performed in the present study. Therefore, associations between the observed histological patterns and graft properties such as porosity, scaffold architecture, or degradation behavior should be regarded as interpretative hypotheses rather than as mechanisms directly demonstrated by the present experiment.

### 2.8. Anesthetic Protocol

Each rabbit was premedicated with acepromazine at 1.0 mg/kg IM (Acepran^®^, Vetnil; Louveira, SP, Brazil). Fifteen minutes later, general anesthesia was induced by the intramuscular administration of xylazine at 3.0 mg/kg (Dopaser^®^, Hertape Calier; Juatuba, MG, Brazil) combined with ketamine hydrochloride at 50.0 mg/kg (União Química Farmacêutica Nacional S/A; Embu-Guaçu, SP, Brazil). Once an adequate level of anesthesia had been achieved, oxytetracycline was administered intramuscularly at 0.2 mL/kg for antibiotic prophylaxis (Biovet; Vargem Grande Paulista, SP, Brazil).

The surgical field was subsequently clipped and prepared with a 1% povidone–iodine solution (Riodeíne^®^ Tincture, Rioquímica; São José do Rio Preto, SP, Brazil). Additional local anesthesia was obtained by infiltrating 2% mepivacaine containing norepinephrine at a concentration of 1:100,000 (Mepinor^®^, Nova DFL; Rio de Janeiro, RJ, Brazil).

### 2.9. Surgical Procedures

**Figure 2:**
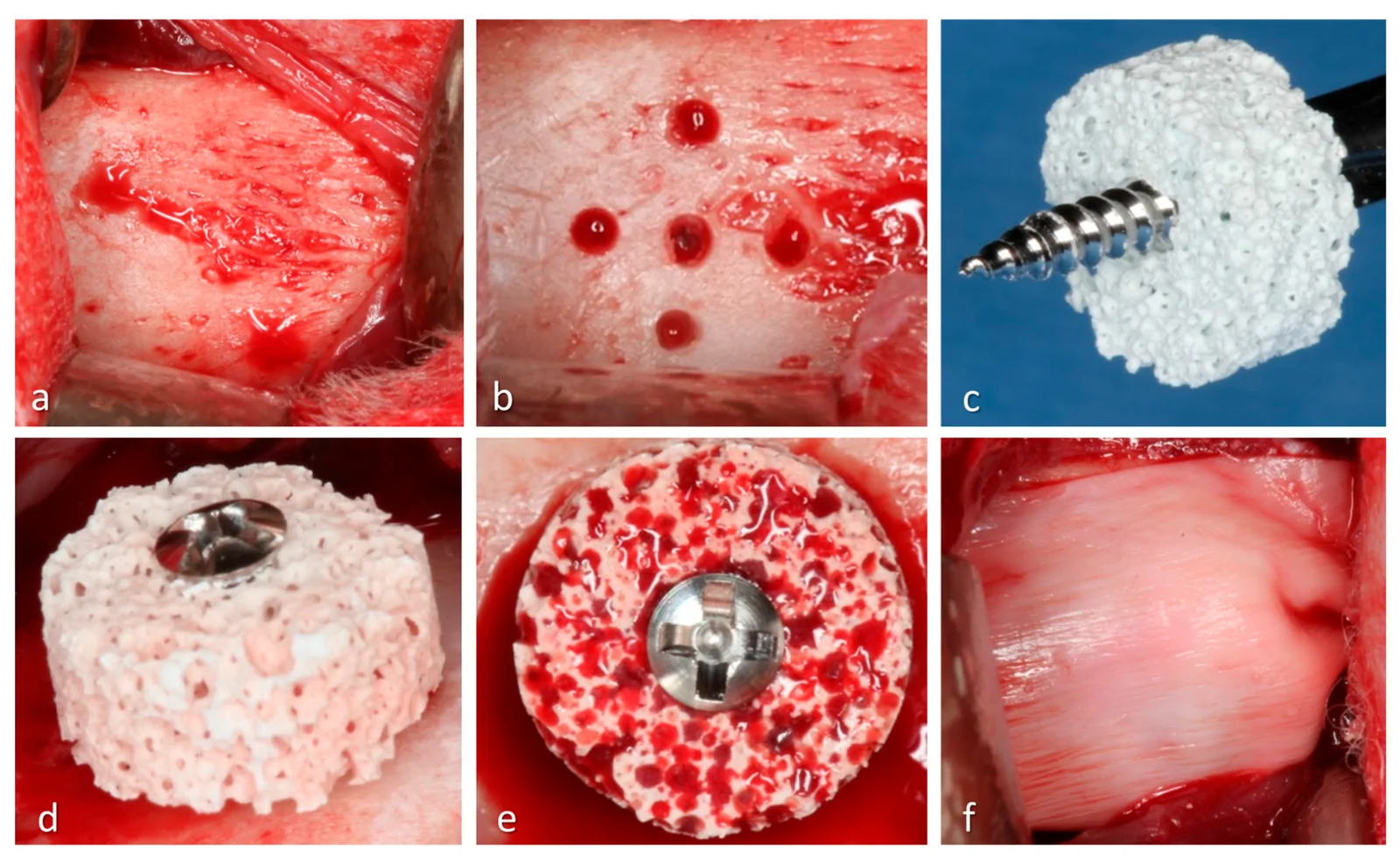
Surgical procedure for onlay block augmentation on the lateral aspect of the rabbit mandible. (**a**) Exposure of the lateral surface of the mandibular angle after reflection of the soft tissues and periosteum. (**b**) Preparation of five monocortical perforations in the recipient bed to access the underlying medullary compartment. (**c**) Biomaterial block with the titanium fixation screw positioned through the central perforation. (**d**) Block immediately after placement and fixation, before appreciable blood infiltration. (**e**) Appearance after blood had penetrated the porous structure of the block, filling the interconnected pores and cavities. (**f**) Coverage of the augmented site with a resorbable collagen membrane.

All surgeries were performed by a single experienced operator (E.R.S.). A linear skin incision of approximately 2.5–3.0 cm was made bilaterally along the lower border of the mandible. The muscles and periosteum were carefully reflected to expose the lateral surface of the mandibular angle (Figure 2).

At each experimental site, the cortical recipient bed was prepared with five equidistant monocortical perforations using a 1.0 mm truncated-conical drill mounted on a straight handpiece under constant sterile saline irrigation. The perforations were performed using a stainless-steel template and extended to the medullary compartment to promote blood supply and cellular migration from the endosteal region.

Each biomaterial block was adapted to the prepared recipient bed. A central perforation was prepared through the block to allow insertion of the fixation screw. The block was positioned in an onlay configuration on the lateral surface of the mandible and fixed using a 1.5 × 10 mm titanium screw (Neodent, Curitiba, Brazil). After fixation, the experimental region was covered with an OsteoBiol^®^ Evolution collagen membrane.

The muscle layers were repositioned and sutured using Vicryl 4-0 (Ethicon, Johnson & Johnson, Cincinnati, OH, USA). The skin was closed with Nylon 4-0 (Ethicon, Johnson & Johnson, USA) using simple interrupted sutures.

### 2.10. Postoperative Care, Welfare Monitoring, and Humane Endpoints

During the first three postoperative days, the animals received ketoprofen at 3.0 mg/kg every 12 h intramuscularly (Ketofen 10%, Merial, Campinas, São Paulo, Brazil) and tramadol hydrochloride 2% at 1.0 mg/kg every 12 h subcutaneously (Cronidor, Agener União Saúde Animal, Apucarana, Paraná, Brazil).

The animals underwent daily welfare checks performed jointly by the investigators, veterinary personnel, and animal-care staff. Monitoring focused on feeding and drinking behavior, urine and fecal production, activity and general behavior, clinical signs of discomfort or distress, overall health, and the condition of the surgical site, including edema, hemorrhage, wound separation, or signs suggestive of infection.

Criteria for early withdrawal were established before the start of the experiment. These included sustained loss of appetite or inadequate hydration, pain not adequately controlled by the prescribed analgesic regimen, serious infection, wound breakdown likely to interfere with healing, pronounced locomotor impairment, progressive worsening of the animal’s condition, or any other clinical situation judged incompatible with continued participation. When one of these conditions was detected, the rabbit was promptly assessed by the responsible veterinarian and, if a satisfactory recovery was considered improbable, euthanasia was performed in accordance with institutional procedures.

### 2.11. Euthanasia and Specimen Collection

After 10 weeks of healing, the animals were euthanized by administration of an overdose of intravenous thiopental 1.0 g, 2.0 mL (Thiopentax, Cristália, Itapira, São Paulo, Brazil), in accordance with the guidelines of the Animal Ethics Committee.

The experimental regions were carefully dissected and reduced into individual blocks containing the grafted region, the fixation screw, and the surrounding native bone. All specimens were fixed in 10% paraformaldehyde for 10 days, with regular changes of the fixative solution every two days.

### 2.12. Histological Processing

After fixation, the specimens were rinsed under running water to remove residual fixative solution. The samples were dehydrated in an ascending series of ethyl alcohol solutions, changed every three days under constant agitation: 60%, 80%, 96%, and absolute alcohol twice.

The specimens were then infiltrated and embedded in LR White™ HardGrid resin (London Resin Co. Ltd., Reading, Berkshire, UK). Polymerization was performed in an oven at 60 °C.

After polymerization, each specimen was sectioned through the center of the grafted region, using the fixation screw as the main reference landmark. One undecalcified ground section per experimental site, approximately 100–150 μm thick, was obtained using a precision cutting/grinding system (Exakt, Apparatebau, Norderstedt, Germany). Each section was then ground to a final thickness of approximately 60–80 μm and stained with Stevenel’s blue and alizarin red.

### 2.13. Histomorphometric Outcomes and Measurements

Photomicrographs were obtained using 1.6×, 10×, and 40× objectives on a trinocular upright microscope equipped for bright-field and polarized-light microscopy (DMLB; Leica Microsystems, Wetzlar, Germany). Images acquired using the 10× objective were used for the histomorphometric measurements. All measurements were performed using ImageJ 1.54g image-analysis software (National Institutes of Health, Bethesda, MD, USA).

**Figure 3:**
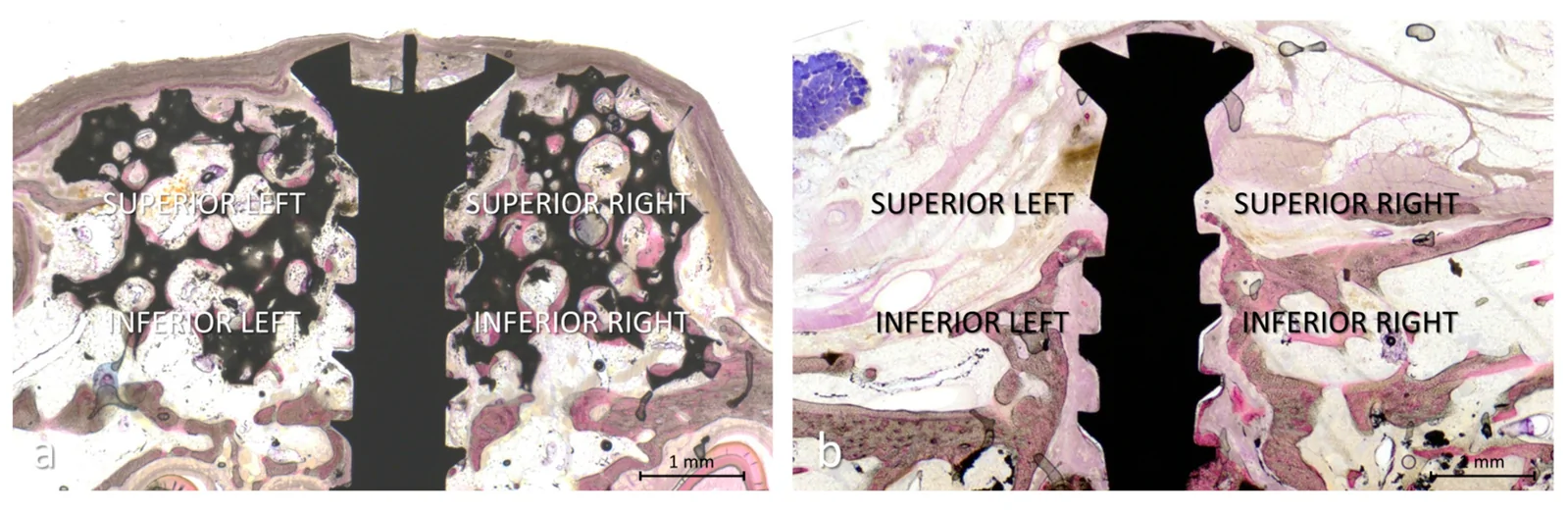
Representative histological sections illustrating the standardized location of the superior and inferior regions used for histomorphometric analysis. The labels indicate the regions corresponding to the four standardized regions of interest positioned on both sides of the fixation screw. (**a**) Representative specimen with substantial preservation of the augmented compartment, in which the standardized regions remained within the residual grafted/regenerated area. (**b**) Representative specimen showing marked reduction in the augmented compartment, resulting in portions of the standardized regions extending beyond the remaining hard-tissue contour. The position of the regions was determined according to the recipient bone surface and fixation screw, independently of the final dimensions of the augmented compartment. Stevenel’s blue and alizarin red stain.

For each specimen, four standardized rectangular regions of interest were selected within the expected original grafted area (Figure 3a,b). Each region measured 2 mm × 1 mm. Two rectangles were positioned on one side of the fixation screw and two on the opposite side. On each side, one rectangle was placed in the inferior region, immediately above the mandibular recipient bed, and the other in the superior region, toward the external surface of the original block. The inferior border of the lower regions was aligned with the recipient bone surface without including native bone.

The position of the regions of interest was standardized according to the recipient bone surface and fixation screw, independently of the final dimensions of the residual grafted or regenerated compartment. Consequently, in specimens showing marked graft contraction or resorption, portions of the standardized regions could extend beyond the remaining hard-tissue contour.

A point-counting method was applied by superimposing a lattice grid on each region of interest (ROI) using squares 75 µm in dimension. Each lattice intersection was assigned to one, and only one, of the following mutually exclusive categories:

- Newly formed bone;
- Residual graft;
- IBN-like tissue;
- Intra-compartment soft tissue;
- Extra-compartment soft tissue;
- Other components.

A lattice intersection was classified as IBN-like only when newly formed bone and residual biomaterial were both visually identifiable at the same counting point but could not be reliably assigned to separate categories because they were closely intermingled or optically superimposed. Classification was performed using standardized transmitted-light conditions, followed, when necessary, by increased light intensity and adjustment of the focal plane. When a counting point could be unequivocally assigned to either newly formed bone or residual graft, it was recorded in the corresponding conventional category rather than as IBN-like.

Intra-compartment soft tissue included non-mineralized tissue located within the external contour of the residual grafted or regenerated compartment and not assigned to any of the other specifically identified categories.

Extra-compartment soft tissue included non-mineralized tissue located within the standardized region of interest (ROI) but outside the residual grafted or regenerated hard-tissue contour. Because the regions of interest were positioned according to fixed anatomical references rather than the final dimensions of the regenerated compartment, this category represented the proportion of the standardized region no longer occupied by the residual grafted or regenerated compartment and was therefore interpreted primarily as an indicator of compartment contraction or resorption.

Other components included vascular structures, inflammatory infiltrate, residual collagen membrane, muscle fibers, and osteoblast-rich areas. These structures were grouped because each represented a relatively limited and variably distributed proportion of the standardized regions and was not considered a separate primary histomorphometric outcome.

For each histological section, the two inferior regions of interest, located on opposite sides of the fixation screw, were averaged to obtain a single inferior value. Similarly, the two superior regions of interest were averaged to obtain a single superior value. The resulting inferior and superior values were used as the site-level values for each histomorphometric outcome. The overall site-level value was calculated as the mean of the inferior and superior site-level values. Thus, each hemimandibular experimental site contributed one inferior, one superior, and one overall value for each outcome.

### 2.14. Cross-Sectional Area of the Augmented Region

The same ground section used for the histomorphometric analysis was used to measure the cross-sectional area of the augmented region. The area was measured planimetrically on calibrated digital images using ImageJ 1.54g software. The external contour of the residual grafted or regenerated compartment was manually delineated, using the surface of the mandibular recipient bed as the basal boundary. The traced area comprised the entire space enclosed within this contour, including newly formed bone, residual biomaterial, IBN-like tissue, non-mineralized tissue, and the portion of the fixation screw located within the augmented compartment. Tissues located outside this contour were excluded. The resulting area was expressed in square millimeters (mm^2^) and was used solely as a two-dimensional indicator of the extent of the augmented region at 10 weeks. It was not interpreted as a direct measurement of augmented volume or volumetric stability.

### 2.15. Statistical Analysis

Site-level data were summarized as mean ± standard deviation. The percentage of newly formed bone within the standardized regions of interest was designated as the primary histomorphometric outcome. Secondary outcomes included the percentages of residual graft, IBN-like tissue, intra-compartment soft tissue, extra-compartment soft tissue, and other components, as well as the cross-sectional area of the augmented region. Comparisons between the inferior and superior regions were considered exploratory regional analyses.

Bio-Oss^®^ Block was used as the reference material because deproteinized bovine bone mineral is extensively used as a xenogeneic scaffold in bone-augmentation procedures and is characterized by a slow resorption profile. The main planned comparisons therefore evaluated each test biomaterial against Bio-Oss^®^ Block.

The hemimandibular site was considered the experimental unit. Because each rabbit contributed two experimental sites, observations obtained from the same animal were treated as correlated rather than fully independent. Moreover, site allocation was not constrained to ensure that each rabbit received two different biomaterials.

For overall site-level outcomes suitable for mixed-model analysis, comparisons among biomaterials were performed using linear mixed-effects models, with biomaterial included as a fixed factor and animal as a random intercept. Degrees of freedom were estimated using the Satterthwaite approximation. Planned contrasts comparing each test biomaterial with Bio-Oss^®^ Block were estimated from the mixed model and adjusted for multiple comparisons using Dunnett’s method.

For the regional analyses, biomaterial, region (inferior or superior), and their interaction were included as fixed factors. Animal and experimental site nested within animal were included as random effects to account for the correlation between the two sites from the same rabbit and between the inferior and superior measurements obtained from the same site. Comparisons between each test material and Bio-Oss^®^ Block were performed separately within the inferior and superior regions using Dunnett-adjusted contrasts. Inferior–superior comparisons within each material were adjusted using Holm’s method.

Because IBN-like tissue was absent from all Bio-Oss^®^ Block and SP-Block sites and was present in all ReproBone^®^ Block sites but only one Alos Block site, its distribution showed structural zeros and marked separation among materials. Extra-compartment soft tissue showed a similarly sparse distribution, being absent from the Bio-Oss^®^ Block, SP-Block, and ReproBone^®^ Block groups and present almost exclusively in the Alos Block group. These two outcomes were therefore summarized descriptively and were not analyzed using standard Gaussian mixed-effects models.

Model assumptions were evaluated by inspecting residual-versus-fitted plots and quantile-quantile plots of the residuals. The Shapiro–Wilk test was used only as a supplementary assessment and was not interpreted in isolation. When material deviations from model assumptions were identified, an appropriate transformation was considered. If an adequate model could not be obtained, the corresponding outcome was summarized descriptively and interpreted as exploratory rather than being analyzed using rank-based tests that did not account for within-animal clustering.

The statistical significance level was set at 5%. Multiplicity adjustment was applied to the planned comparisons between each test biomaterial and Bio-Oss^®^ Block within each outcome. Because the secondary and regional outcomes were exploratory, their results were interpreted cautiously.

Statistical analyses were performed using R software, version 4.6.1 (R Foundation for Statistical Computing, Vienna, Austria), with the lme4, lmerTest, and emmeans packages.

## 3. Results

### 3.1. Clinical Outcomes

All animals completed the 10-week healing period without postoperative complications. No animals or experimental sites were excluded from the subsequent analyses.

### 3.2. Qualitative Morphological Evaluation

The four block biomaterials exhibited different patterns of structural preservation, bone ingrowth, and tissue organization after 10 weeks of healing.

**Figure 4:**
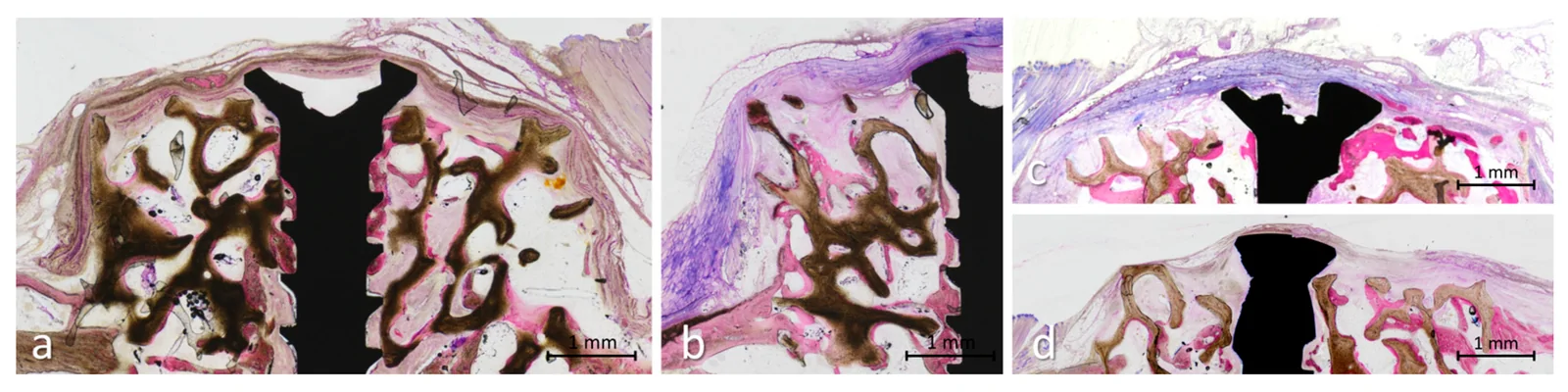
Photomicrographs of histological sections of Bio-Oss^®^ Block after 10 weeks of healing. (**a**) Overview showing substantial preservation of the original block cross-sectional contour and trabecular architecture, with newly formed bone extending from the mandibular recipient bed along the surfaces of the trabeculae of the residual biomaterial and of the screw surface. (**b**) Detail showing newly formed bone forming bridging connections from the base of the mandible along the lateral aspect of the scaffold. (**c**,**d**) Photomicrographs of coronal areas showing newly formed bone reaching the most coronal region of the graft. Stevenel’s blue and alizarin red stain.

In the Bio-Oss^®^ Block group, the overall dimensions of the graft and its trabecular architecture appeared largely preserved. Newly formed bone originated from the recipient mandibular bed and extended toward the superior portion of the block, developing in close association with the surfaces of the biomaterial trabeculae (Figure 4a).

This pattern was readily evident in the inferior region, where newly formed bone progressively lined the residual graft structure and, in some specimens, was also observed in contact with the fixation screw. In selected sections, newly formed bone covered a substantial part of the external aspect of the graft, forming bridging connections between bone originating from the mandibular bed and bone deposited along the biomaterial scaffold (Figure 4b). Bone-to-screw contact was also occasionally observed around the more coronal portion of the fixation screw (Figure 4c,d).

The spaces between the residual biomaterial trabeculae were predominantly occupied by marrow-like tissue showing variable degrees of maturation. Only limited inflammatory cell infiltration and occasional osteoclasts were observed. Remnants of the covering membrane were also identified in some sections.

**Figure 5:**
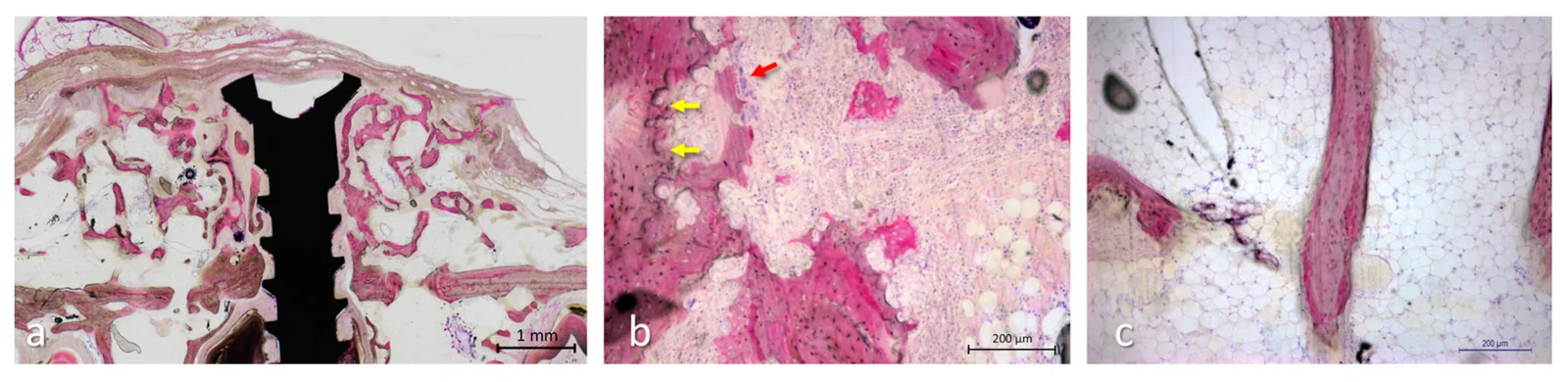
Photomicrographs of histological sections of SP-Block after 10 weeks of healing. (**a**) Overview showing relative preservation of the augmented cross-sectional contour, although a substantial portion of the original trabecular architecture was no longer identifiable. Newly formed bone extended from the mandibular recipient bed into the grafted area and, in some specimens, reached the superior portion of the block. (**b**) Detail showing residual biomaterial trabeculae with Howship’s lacunae (yellow arrows), consistent with previous osteoclastic activity; osteoclast-like cells were only occasionally observed at the 10-week healing period (red arrow). (**c**) Photomicrograph of area showing marrow-like tissue occupying the intervening spaces between residual biomaterial and newly formed bone. Stevenel’s blue and alizarin red stain.

In the SP-Block group, the overall dimensions of the graft appeared relatively well preserved, although a substantial portion of its original trabecular architecture was no longer identifiable (Figure 5a).

Residual biomaterial trabeculae were variably distributed within the grafted area and frequently exhibited irregular surfaces and resorption bays consistent with previous osteoclastic activity.

At the 10-week observation period, however, osteoclasts were only occasionally detected (Figure 5b). Newly formed bone extended from the recipient mandibular bed into the inferior region of the graft and, in some areas, developed along the surfaces of the residual biomaterial trabeculae, indicating that the remaining scaffold continued to support bone ingrowth. In several sections, newly formed bone reached the superior region of the graft, where residual biomaterial fragments were still present while bone formation was rarely observed in contact with the fixation screw (Figure 5a).

The intervening spaces were predominantly occupied by marrow-like tissue, including areas with a more mature, adipocyte-rich bone-marrow appearance, with limited inflammatory cell infiltration and only sparse osteoclasts (Figure 5c). Remnants of the covering membrane were also observed in most of the sections.

**Figure 6:**
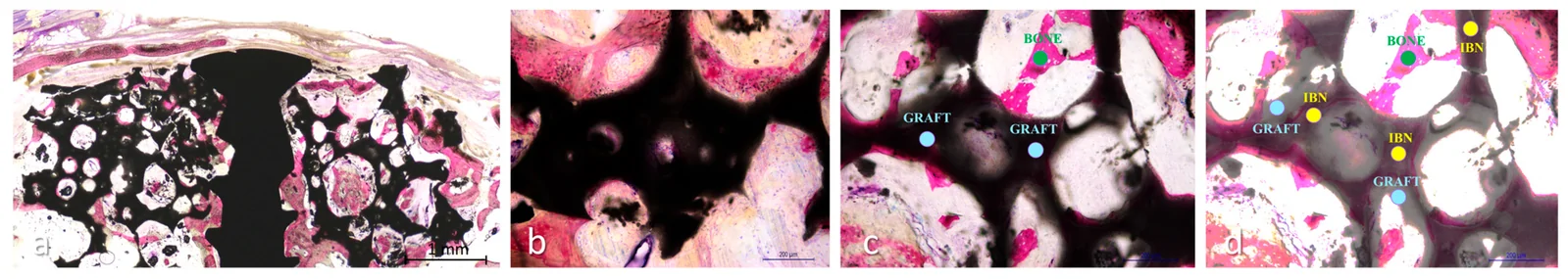
Photomicrographs of histological sections of ReproBone^®^ Block after 10 weeks of healing. (**a**) Overview showing substantial preservation of the augmented cross-sectional contour and of the interconnected porous architecture, with newly formed bone extending from the mandibular recipient bed into the grafted area. (**b**) Detail showing newly formed bone in direct apposition to the surfaces of the residual biomaterial, with marrow-like tissue occupying the remaining pore spaces. (**c**) Representative area in which newly formed mineralized tissue appeared to form a continuous network across adjacent cavities within the biomaterial. (**d**) The same area shown in (**c**), illustrating the interpenetrating bone network-like (IBN-like) pattern, in which newly formed bone appeared closely intermingled with the residual biomaterial. GRAFT, residual biomaterial; BONE, newly formed bone; IBN, interpenetrating bone network-like tissue. Stevenel’s blue and alizarin red stain.

In the ReproBone^®^ Block group, the overall dimensions and porous architecture of the graft appeared largely preserved after 10 weeks of healing, with substantial amounts of residual biomaterial remaining within the augmented area. Newly formed bone extended from the recipient mandibular bed into the inferior region of the graft and progressively occupied its internal porous network (Figure 6a). Within the pores, newly formed bone was frequently observed in direct apposition to the surfaces of the residual biomaterial. The remaining spaces were occupied by marrow-like tissue showing variable degrees of maturation (Figure 6b). In several specimens, newly formed bone reached the superior region of the block and extended along its peripheral portion. In selected sections, newly formed bone was also observed in contact with the fixation screw (Figure 6a).

In some areas, newly formed mineralized tissue appeared to form a continuous network across adjacent cavities within the biomaterial (Figure 6c). Under increased transmitted light, some mineralized areas appeared to develop within, or to be closely intermingled with, the residual biomaterial. This pattern was more clearly recognizable as newly formed bone occupying interconnected pores or cavities located on different planes of the histological section, rather than being confined to conventional bone apposition along a clearly demarcated graft surface. This appearance was classified as an interpenetrating bone network-like (IBN-like) pattern (Figure 6c,d).

**Figure 7:**
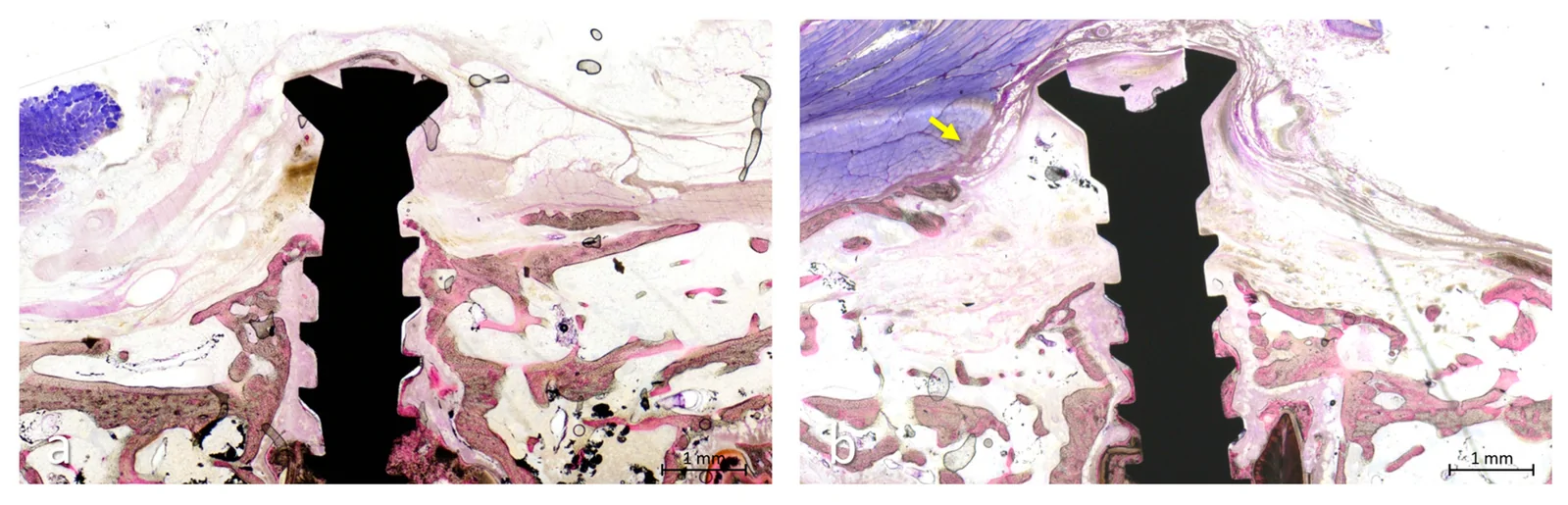
Photomicrographs of histological sections of Alos Block after 10 weeks of healing. (**a**) Overview showing marked loss of the original biomaterial scaffold and poor preservation of the initial block cross-sectional contour, with the superior portion of the augmented area largely occupied by connective tissue. (**b**) Detail showing peripheral mineralized tissue with a more cortical-like organization surrounding marrow-like spaces (yellow arrow) and extending toward the mandibular recipient bed, establishing continuity with the native basal bone. Stevenel’s blue and alizarin red stain.

In the Alos Block group, the original dimensions and architecture of the graft were poorly preserved because of the marked loss of the original biomaterial scaffold (Figure 7a).

The peripheral walls of the regenerated tissue frequently showed a more cortical-like organization and surrounded broad areas of marrow-like tissue. These mineralized walls extended toward the mandibular recipient bed, establishing a direct connection with the native basal bone (Figure 7b). The superior portion of the augmented area was predominantly occupied by connective tissue (Figure 7a,b). Only sparse residual biomaterial particles were present, together with very limited areas showing an IBN-like pattern. Remnants of the covering membrane were also observed in some sections.

### 3.3. Quantitative Morphometric Evaluation

#### 3.3.1. Overall Tissue Composition Within the Standardized Regions of Interest

**Figure 8:**
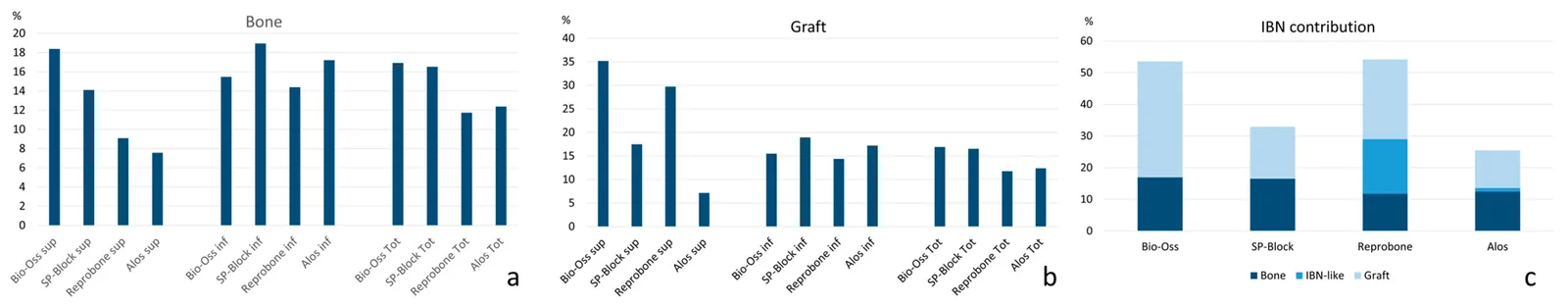
Regional and overall distribution of newly formed bone, residual graft, and IBN-like tissue after 10 weeks of healing. (**a**) Mean percentages of newly formed bone in the superior (sup) and inferior (inf) regions and in the overall standardized region (Tot). (**b**) Corresponding percentages of residual graft. (**c**) Stacked representation of the overall percentages of newly formed bone, IBN-like tissue, and residual graft. IBN-like tissue is positioned between newly formed bone and residual graft to visually illustrate how the mineralized tissue composition would change if IBN-like tissue were considered together with either newly formed bone or residual graft. IBN-like tissue was maintained as a separate morphometric category in all quantitative analyses.

Quantitative morphometric analysis revealed distinct tissue-composition patterns among the four biomaterials after 10 weeks of healing (Table 1; Figure 8a–c).

The mean percentage of newly formed bone was 16.9 ± 3.8% in the Bio-Oss^®^ Block group, 16.5 ± 10.1% in the SP-Block group, 11.7 ± 5.4% in the ReproBone^®^ Block group, and 12.4 ± 7.6% in the Alos Block group. No significant overall difference was detected among the biomaterials (*p* = 0.475), and none of the test materials differed significantly from Bio-Oss^®^ Block in the planned comparisons (all adjusted *p* ≥ 0.466).

Residual graft accounted for 36.7 ± 9.0% of the standardized regions in the Bio-Oss^®^ Block group, 16.3 ± 9.1% in the SP-Block group, 25.2 ± 3.8% in the ReproBone^®^ Block group, and 11.9 ± 4.3% in the Alos Block group. A significant overall difference was found among the biomaterials (*p* < 0.001). Compared with Bio-Oss^®^ Block, the percentage of residual graft was significantly lower in the SP-Block (adjusted *p* < 0.001), ReproBone^®^ Block (adjusted *p* = 0.027), and Alos Block groups (adjusted *p* < 0.001).

IBN-like tissue was absent from all Bio-Oss^®^ Block and SP-Block sites. It was identified in all six ReproBone^®^ Block sites, in which it represented 17.3 ± 2.9% of the standardized regions. In the Alos Block group, IBN-like tissue was detected in only one of the six sites, resulting in a group mean of 1.2 ± 2.9%. Because of the structural zero values and marked material-dependent distribution, IBN-like tissue was evaluated descriptively.

Intra-compartment soft tissue represented 38.4 ± 7.2%, 56.0 ± 13.3%, 41.5 ± 11.3%, and 49.8 ± 9.0% in the Bio-Oss^®^ Block, SP-Block, ReproBone^®^ Block, and Alos Block groups, respectively. The overall material effect was significant (*p* = 0.041). The percentage was significantly higher in the SP-Block group than in the Bio-Oss^®^ Block group (adjusted *p* = 0.026), whereas the other planned comparisons were not significant.

Extra-compartment soft tissue was absent from all Bio-Oss^®^ Block, SP-Block, and ReproBone^®^ Block sites. In the Alos Block group, it was observed in five of the six sites and represented 6.6 ± 6.6% of the standardized regions. This category corresponded to soft tissue located within the standardized regions of interest but outside the remaining hard-tissue contour and was therefore interpreted as a descriptive indicator of contraction or loss of the cross-sectional augmented contour.

Other components represented 8.0 ± 1.5%, 11.1 ± 3.3%, 4.3 ± 2.7%, and 18.2 ± 6.6% in the Bio-Oss^®^ Block, SP-Block, ReproBone^®^ Block, and Alos Block groups, respectively. The overall material effect was significant (*p* = 0.002). Compared with Bio-Oss^®^ Block, a significantly higher percentage was observed only in the Alos Block group (adjusted *p* = 0.013).

#### 3.3.2. Superior and Inferior Regions

Regional analysis identified an overall difference in newly formed bone between the inferior and superior regions and a material-dependent regional distribution of residual graft (Table 2).

When data were averaged across biomaterials, the percentage of newly formed bone differed significantly between the inferior and superior regions (*p* = 0.020). No significant overall material effect (*p* = 0.475) or material-by-region interaction (*p* = 0.094) was detected. The mean inferior and superior values were 15.5% and 18.4% for Bio-Oss^®^ Block, 18.9% and 14.1% for SP-Block, 14.4% and 9.1% for ReproBone^®^ Block, and 17.2% and 7.6% for Alos Block, respectively. None of the test materials differed significantly from Bio-Oss^®^ Block within either region (all adjusted *p* ≥ 0.078).

In the planned comparisons between regions within each biomaterial, newly formed bone was significantly greater in the inferior than in the superior region of the Alos Block group, with an estimated difference of 9.6 percentage points (Holm-adjusted *p* = 0.036). No significant inferior–superior difference was detected within the other biomaterial groups.

Residual graft showed a significant material effect (*p* < 0.001) and a significant material-by-region interaction (*p* = 0.023), whereas no overall regional effect was detected (*p* = 0.896). In the inferior region, residual graft represented 38.2% in the Bio-Oss^®^ Block group, 15.2% in the SP-Block group, 20.6% in the ReproBone^®^ Block group, and 16.6% in the Alos Block group. All three test materials showed significantly lower values than Bio-Oss^®^ Block in this region (adjusted *p* < 0.001, *p* = 0.003, and *p* < 0.001, respectively).

In the superior region, residual graft represented 35.2%, 17.5%, 29.7%, and 7.2% in the Bio-Oss^®^ Block, SP-Block, ReproBone^®^ Block, and Alos Block groups, respectively. Significantly lower values than Bio-Oss^®^ Block were observed for SP-Block (adjusted *p* = 0.003) and Alos Block (adjusted *p* < 0.001), whereas ReproBone^®^ Block did not differ significantly from the reference material (adjusted *p* = 0.550). Despite the numerical differences between the inferior and superior regions, none of the within-material regional contrasts for residual graft remained significant after Holm adjustment (all adjusted *p* ≥ 0.111).

In the ReproBone^®^ Block group, IBN-like tissue represented 16.6% of the inferior region and 18.0% of the superior region. In the Alos Block group, the limited IBN-like component was confined to the superior region, where it represented 2.4%, and originated from a single specimen. Extra-compartment soft tissue in the Alos Block group was also predominantly located in the superior region, accounting for 13.0%, compared with 0.2% in the inferior region.

No significant overall regional effect or material-by-region interaction was detected for intra-compartment soft tissue or other components.

#### 3.3.3. Cross-Sectional Area of the Augmented Region

The mean cross-sectional area of the augmented region was 24.1 ± 2.1 mm^2^ in the Bio-Oss^®^ Block group, 23.1 ± 6.2 mm^2^ in the SP-Block group, 22.9 ± 2.0 mm^2^ in the ReproBone^®^ Block group, and 12.9 ± 6.1 mm^2^ in the Alos Block group.

The overall difference among biomaterials was significant (*p* = 0.001). Compared with Bio-Oss^®^ Block, the augmented area was significantly smaller in the Alos Block group (adjusted *p* = 0.001). No significant differences were found between Bio-Oss^®^ Block and either SP-Block or ReproBone^®^ Block.

Taken together, the quantitative findings showed comparable percentages of newly formed bone among the four biomaterials but marked differences in scaffold persistence, mode of incorporation, tissue organization, and cross-sectional augmented area at 10 weeks. Bio-Oss^®^ Block retained the greatest proportion of conventionally recognizable residual graft. ReproBone^®^ Block was characterized by a substantial and consistently observed IBN-like component. Alos Block showed the smallest augmented area, the lowest persistence of residual graft, and the greatest representation of soft tissue outside the remaining augmented contour.

## 4. Discussion

### 4.1. Principal Findings and Relevance of the Onlay Model

The present exploratory study compared the healing and remodeling patterns of two xenogeneic and two synthetic block biomaterials used as onlay grafts on the lateral aspect of rabbit mandibles. After 10 weeks of healing, all four materials supported new bone formation, with no significant differences in the overall percentage of newly formed bone. Nevertheless, marked differences were observed in scaffold persistence, tissue organization, mode of incorporation, and cross-sectional area observed at 10 weeks. These findings indicate that the biological performance of block biomaterials cannot be interpreted solely according to the percentage of newly formed bone but requires consideration of the residual scaffold, soft-tissue organization, IBN-like tissue, and cross-sectional augmented area at 10 weeks.

These differences are particularly relevant in an onlay model. Unlike an inlay graft placed within a contained defect, an onlay block has limited contact with native bone and therefore depends largely on vascular and osteogenic ingrowth from the underlying recipient bed, although the repositioned periosteum may provide an additional contribution from the outer aspect. In the present model, the collagen membrane was interposed between the block and the repositioned periosteum and could therefore have modified or delayed the periosteal contribution, particularly during early healing. Experimental evidence suggests that this effect may depend on the membrane and experimental model: a non-cross-linked collagen membrane did not substantially impair periosteal regenerative capacity in a rat calvarial model, whereas lower neoangiogenesis was observed beneath a resorbable barrier membrane than beneath a rapidly resorbing collagen fleece in rabbit calvarial defects [39,40]. In the present study, all sites received the same membrane, and neither a membrane-free control nor direct measurements of vascularization were included. Consequently, whether the membrane reduced revascularization or de novo osteogenesis cannot be determined. The cortical perforations were nevertheless intended to promote vascular and cellular supply from the underlying medullary compartment.

Bone formation must therefore progress from the mandibular surface toward regions increasingly distant from the native bone, while the graft must simultaneously resist soft-tissue pressure and preserve the intended augmentation contour. Previous experiments using the same rabbit mandibular model showed that inlay grafts generally provide more favorable conditions for bone formation because they are exposed to multiple surrounding bone surfaces [23,24]. Nevertheless, the present study demonstrated that newly formed bone could also reach the superior regions of onlay blocks when a stable and sufficiently persistent osteoconductive framework was maintained.

The absence of a significant material-by-region interaction for newly formed bone suggests that its regional distribution was not consistently different among materials. However, a significantly greater percentage of newly formed bone was observed in the inferior than in the superior region of the Alos Block group. Conversely, residual graft showed a significant material-by-region interaction, indicating that scaffold persistence differed not only among biomaterials but also according to position within the block. Overall, the findings suggest that onlay graft healing may be influenced by the balance among scaffold persistence, osteoconductive continuity, and tissue formation. Because the materials were not independently characterized and only one healing interval was evaluated, explanations involving scaffold architecture and degradation kinetics should be regarded as interpretative rather than mechanistically demonstrated.

### 4.2. Bio-Oss^®^ Block: Structural Persistence and Surface-Guided Osteoconduction

Bio-Oss^®^ Block showed the greatest proportion of residual graft and maintained a cross-sectional augmented area comparable to those of SP-Block and ReproBone^®^ Block. Despite the persistence of the scaffold, newly formed bone was not confined to the interface with the mandibular recipient bed. Bone developed along the surfaces of the residual trabeculae and, in several specimens, reached the superior and peripheral portions of the block. This pattern was characterized by preservation of a continuous trabecular framework, with bone growth occurring along its surfaces.

However, scaffold persistence should not be interpreted as biological replacement. Newly formed bone was frequently deposited as relatively thin layers along the residual biomaterial surfaces, and the overall percentage of bone did not differ significantly from that observed with the other materials. Thus, preservation of the cross-sectional augmented area at 10 weeks and extensive spatial distribution of bone did not necessarily correspond to replacement of the graft by vital tissue.

Previous canine studies reported more limited incorporation of DBBM blocks, with bone formation mainly restricted to the basal region or connective tissue interposed between the graft and the recipient bed [1,21]. These differences cannot be explained solely by the onlay or inlay configuration. Graft adaptation, uninterrupted stability at the recipient interface, vascular access, species-related healing dynamics, and the surgical procedures performed during healing may also influence incorporation.

In the present study, the blocks remained undisturbed throughout the 10-week healing period. In contrast, in the experiments by De Santis et al. [21], implant osteotomies were subsequently prepared at the interface between the graft and native bone after only 3 months of healing. This relatively short healing interval may also have contributed to the observed outcome, as the newly established connections between the graft and native bone might not yet have reached sufficient maturity. Although this remains speculative, drilling and implant insertion may therefore have disrupted immature connections or displaced the brittle scaffold. Overall, Bio-Oss^®^ Block showed preservation of the cross-sectional augmented area and a surface-guided pattern of osteoconduction at 10 weeks, while the substantial amount of residual scaffold indicated limited biological substitution during the evaluated interval.

### 4.3. SP-Block: Heterogeneous Scaffold Remodeling

SP-Block showed significantly less residual graft than Bio-Oss^®^ Block while maintaining a comparable cross-sectional augmented area. The original trabecular architecture was variably preserved, and residual trabeculae frequently displayed irregular surfaces and resorption bays (Howship’s lacunae). Newly formed bone developed along the remaining scaffold and occasionally reached the superior region, but its percentage and spatial distribution varied considerably among specimens.

The reduced persistence of the biomaterial was accompanied by a greater proportion of intra-compartment non-mineralized tissue, much of which exhibited a marrow-like appearance, rather than by a significantly greater percentage of newly formed bone. Thus, the lower proportion of recognizable residual graft was not accompanied by a proportional increase in mineralized bone, although part of the space created during remodeling appeared to undergo maturation toward bone marrow rather than being occupied simply by undifferentiated connective tissue. When loss of the trabecular framework occurred before sufficient bone deposition, however, the continuity of the mineralized osteoconductive pathways toward the superior region may have been reduced.

The sparse osteoclasts observed at 10 weeks, together with the presence of Howship’s lacunae, suggest that more active resorption may have occurred earlier during healing. Effective remodeling requires coordination between osteoclastic resorption and subsequent bone formation, whereas an imbalance between these processes may result in net loss of the mineralized scaffold [41]. SP-Block therefore showed a more pronounced and heterogeneous remodeling pattern than Bio-Oss^®^ Block, characterized by reduced graft persistence, new bone formation, and marrow maturation.

### 4.4. ReproBone^®^ Blocks: Interconnected Porosity and IBN-like Pattern

ReproBone^®^ Blocks maintained a cross-sectional augmented area comparable to that of Bio-Oss^®^ Block, despite showing a significantly lower proportion of conventionally recognizable residual graft. Newly formed bone was observed at different levels of the porous scaffold and reached the superior regions in several specimens. However, the overall percentage of newly formed bone did not differ significantly from that observed with Bio-Oss^®^ Block or the other materials.

The most distinctive finding was the consistent presence of IBN-like tissue in all ReproBone^®^ specimens. These areas represented a close intermingling or optical superimposition of newly formed bone and residual calcium phosphate scaffold, rather than the clearly demarcated surface apposition typically observed around Bio-Oss^®^ trabeculae. Similar patterns have been described with calcium phosphate biomaterials in experimental sinus augmentation and human biopsies [42,43]. A comparable IBN-like pattern was also observed with ReproBone^®^ novo Paste in a rabbit sinus augmentation model, despite the different anatomical environment and material configuration [44].

Nevertheless, IBN-like tissue should not be interpreted as pure newly formed bone or as evidence that bone penetrated directly through the solid scaffold matrix. In two-dimensional ground sections, bone occupying interconnected pores or structures located at different depths may appear continuous across residual biomaterial. Maintaining IBN-like tissue as a separate category therefore avoided overestimating either bone formation or graft persistence.

Overall, ReproBone^®^ Blocks showed preservation of the cross-sectional augmented area and a frequent histological pattern in which newly formed bone and residual scaffold could not be reliably separated in two-dimensional sections. This IBN-like pattern should be regarded solely as a descriptive observation. Its biological maturation, mechanical competence, long-term fate, and potential relevance to implant osseointegration remain unknown.

### 4.5. Alos Block: Extensive Remodeling and Reduced Cross-Sectional Area

Alos Block showed the lowest proportion of residual graft and the smallest cross-sectional augmented area among the materials evaluated. Although the percentage of newly formed bone did not differ significantly from that observed in the other groups, the original scaffold architecture was largely lost, and the healing pattern varied considerably among specimens.

In some sites, scaffold degradation was accompanied by the formation of organized bone surrounding marrow-like compartments and extending toward the superior region. In others, bone remained predominantly confined to the inferior portion, while the superior region was replaced by connective or muscular tissue. This heterogeneous pattern was also reflected in the regional analysis, in which the percentage of newly formed bone was significantly higher in the inferior than in the superior region of the Alos Block group. Extra-compartment soft tissue was located predominantly in the superior region, a descriptive finding consistent with incomplete maintenance of the original augmentation contour.

Only limited IBN-like tissue was identified, coinciding with extensive scaffold loss and few residual areas in which bone and biomaterial could still be recognized simultaneously. Overall, Alos Block showed the greatest loss of recognizable residual scaffold; however, this was not accompanied by a greater percentage of newly formed bone, and maintenance of the cross-sectional augmented area was less consistent. These observations are compatible with pronounced remodeling, but they do not establish degradation kinetics or long-term material persistence.

A similarly pronounced degradation pattern was observed with Alos Granular in rabbit sinus augmentation, where extensive scaffold degradation was associated with marked reduction in the augmented compartment [44]. However, IBN-like tissue was more evident with the granular formulation than with the Alos Block evaluated in the present study, suggesting that material configuration and the anatomical environment may influence the relationship between scaffold degradation and newly formed bone.

### 4.6. Translational Considerations

From a translational perspective, an ideal block biomaterial would provide initial structural support, permit vascular and bone ingrowth, and remodel in coordination with new bone formation. However, the present exploratory study was not designed to establish clinical performance or to determine the optimal biomaterial for a specific type of defect.

Within the limitations of this model, the findings suggest that the percentage of newly formed bone alone may not fully describe the histological healing pattern of a block biomaterial. Residual scaffold, spatial distribution of bone, tissue organization, IBN-like tissue, and cross-sectional augmented area at 10 weeks provided complementary information. Whether these histological findings translate into three-dimensional maintenance, mechanical competence, implant osseointegration, or performance under functional loading remains unknown.

Autogenous bone remains the biological reference for onlay augmentation because it provides vital, physiologically remodelable tissue. This advantage must be balanced against donor-site morbidity, limited availability, increased surgical time, and postoperative discomfort [13,14]. Substitute blocks avoid graft harvesting and provide standardized, readily available scaffolds, but the present findings do not demonstrate biological equivalence to autogenous bone.

Previous animal experiments provide relevant context for this comparison. In a rabbit mandibular onlay model, autogenous iliac-crest blocks and equine xenogeneic blocks yielded similar percentages of newly formed bone after 60 days; however, the autogenous blocks showed better incorporation into the recipient bed, greater bone volume, and superior overall quality of the grafted area [45]. Similarly, in dogs, autogenous mandibular blocks became vital and integrated with the parent bone, whereas deproteinized bovine bone mineral blocks showed limited bone ingrowth [21]. Nevertheless, these historical comparisons cannot replace a contemporaneous autogenous control because of differences in experimental models, biomaterials, surgical procedures, and healing intervals.

Accordingly, no recommendation regarding clinical material selection or superiority can be made on the basis of this experiment. Direct extrapolation to human lateral ridge augmentation should be made cautiously, and longer-term studies incorporating three-dimensional volumetric analysis, mechanical assessment, implant placement, and functional loading are required.

### 4.7. Study Limitations

Several limitations should be considered when interpreting the present findings. First, this was an exploratory preclinical study performed in rabbit mandibles, and the observed biological response cannot be directly extrapolated to human lateral ridge augmentation. Rabbits exhibit substantially faster bone turnover and healing than humans, and differences in mandibular anatomy, bone characteristics, local mechanical conditions, and the experimental environment should be considered when translating these findings to the clinical setting [46,47]. Moreover, only one healing interval of 10 weeks was evaluated; therefore, the study provides a cross-sectional representation of graft incorporation and remodeling but does not establish their temporal progression.

Second, the sample size was limited. Although the allocation provided six sites for each biomaterial, it was not constrained within animals, and some rabbits received the same material bilaterally. Consequently, comparisons among biomaterials were not uniformly paired within animals. Because no reliable prior estimate of the within-animal correlation was available, the approximate sample-size calculation assumed independent site-level observations and did not account for clustering of the two sites within each rabbit. The calculation may therefore have overestimated the effective statistical power. Although the mixed-effects models accounted for correlation between sites belonging to the same rabbit, the study remains primarily suited to identifying relatively large differences. In addition, complete masking of the examiner could not be maintained because the biomaterials had distinctive histological appearances.

Third, no autogenous bone control was included. Comparisons with previous studies of autogenous block grafts remain indirect because of differences in experimental models, surgical procedures, and healing intervals. The present experiment therefore allows comparison among the four substitute biomaterials but cannot determine whether any of them approaches the biological performance of an autogenous onlay graft under identical conditions.

Fourth, the histomorphometric assessment was based on ground sections and standardized regions of interest. Although this approach allowed consistent comparison of inferior and superior regions and identification of tissues located outside the remaining augmented contour, it cannot fully represent the three-dimensional distribution of bone, residual scaffold, pores, and soft tissues. The cross-sectional area measurements provided complementary information on cross-sectional augmented area at 10 weeks. No micro-computed tomographic or other volumetric assessment was available; therefore, three-dimensional maintenance or volumetric stability could not be determined.

Particular caution is required when interpreting IBN-like tissue. Its identification depended on the recognition of newly formed bone closely intermingled with, or optically superimposed on, residual biomaterial. Section thickness, optical density, overlapping pores, and structures located at different depths may have influenced its classification. Although predefined criteria and standardized illumination were used, IBN-like tissue remains a descriptive histological category rather than a validated biological entity and cannot be considered equivalent to either pure vital bone or residual graft.

The physicochemical and microstructural characteristics of the specific biomaterial batches were not independently assessed. Interpretations linking the observed histological behavior to material composition, porosity, scaffold architecture, or degradation characteristics were therefore based on manufacturer information and published evidence and should be considered hypothesis-generating.

Finally, the study did not assess the mechanical properties of the regenerated regions or their ability to support implant placement and functional loading. The behavior of the different scaffolds during drilling, their potential for fracture or displacement, and subsequent implant osseointegration therefore remain unknown. Future studies should include longer healing periods, three-dimensional volumetric analyses, mechanical assessment, and implant placement.

## 5. Conclusions

In this exploratory rabbit onlay model, all four block biomaterials supported new bone formation after 10 weeks, with no significant differences in the overall percentage of newly formed bone. Nevertheless, they exhibited distinct patterns of scaffold persistence, tissue organization, mode of incorporation, and cross-sectional augmented area at 10 weeks. Bio-Oss^®^ Block showed the greatest persistence of conventionally recognizable residual graft and predominantly surface-guided bone apposition. SP-Block maintained a cross-sectional augmented area comparable to that of Bio-Oss^®^ Block but underwent more pronounced and heterogeneous remodeling, accompanied by a greater proportion of intra-compartment soft tissue. ReproBone^®^ Blocks also maintained a comparable cross-sectional augmented area and consistently exhibited a substantial proportion of tissue classified descriptively as IBN-like. In contrast, Alos Block showed the greatest scaffold loss, the smallest sectional augmented area, and the most frequent presence of soft tissue outside the remaining augmented contour. The scaffold loss was not accompanied by a greater percentage of newly formed bone.

These findings indicate that the performance of block biomaterials cannot be assessed solely according to the percentage of newly formed bone. Residual scaffold, mode of bone incorporation, IBN-like tissue, soft-tissue organization, and cross-sectional augmented area at 10 weeks should be considered together. No material can be regarded as clinically superior on the basis of the present experiment. Longer-term studies incorporating three-dimensional volumetric analysis, mechanical assessment, and implant placement are required to determine the clinical relevance of the observed healing patterns.

## Figures and Tables

**Table 1:** Overall tissue composition within the standardized regions of interest after 10 weeks of healing. Data are expressed as mean ± standard deviation (%). Bone, newly formed bone; Graft, residual biomaterial; IBN-like, interpenetrating bone network-like tissue; Intra-soft, soft tissue located within the remaining hard-tissue contour; Extra-soft, soft tissue located within the standardized region of interest but outside the remaining hard-tissue contour; Other, components not assigned to the preceding categories. *n* = 6 sites per biomaterial.

	Bone	Graft	IBN-like	Intra-Soft	Extra-Soft	Other
Bio-Oss	16.9 ± 3.8	36.7 ± 9.0	0.0 ± 0.0	38.4 ± 7.2	0.0 ± 0.0	8.0 ± 1.5
SP-Block	16.5 ± 10.1	16.3 ± 9.1	0.0 ± 0.0	56.0 ± 13.3	0.0 ± 0.0	11.1 ± 3.3
ReproBone	11.7 ± 5.4	25.2 ± 3.8	17.3 ± 2.9	41.5 ± 11.3	0.0 ± 0.0	4.3 ± 2.7
Alos	12.4 ± 7.6	11.9 ± 4.3	1.2 ± 2.9	49.8 ± 9.0	6.6 ± 6.6	18.2 ± 6.6

**Table 2:** Regional tissue composition within the standardized regions of interest after 10 weeks of healing. (**A**) Superior region. (**B**) Inferior region. Data are expressed as mean ± standard deviation (%). Bone, newly formed bone; Graft, residual biomaterial; IBN-like, interpenetrating bone network-like tissue; Intra-soft, soft tissue located within the remaining hard-tissue contour; Extra-soft, soft tissue located within the standardized region of interest but outside the remaining hard-tissue contour; Other, components not assigned to the preceding categories. *n* = 6 sites per biomaterial. Inferential analyses and adjusted pairwise comparisons are reported in the text.

**(A) Superior**	**Bone**	**Graft**	**IBN-like**	**Intra-soft**	**Extra-soft**	**Other**
Bio-Oss	18.4 ± 7.1	35.2 ± 10.9	0.0 ± 0.0	39.1 ± 8.3	0.0 ± 0.0	7.3 ± 2.8
SP-Block	14.1 ± 11.1	17.5 ± 7.8	0.0 ± 0.0	56.8 ± 11.0	0.0 ± 0.0	11.6 ± 4.7
ReproBone	9.1 ± 4.5	29.7 ± 7.6	18.0 ± 4.5	39.2 ± 12.7	0.0 ± 0.0	4.0 ± 2.5
Alos	7.6 ± 5.7	7.2 ± 6.6	2.4 ± 5.8	49.7 ± 16.6	13.0 ± 12.9	20.2 ± 12.2
**(B) Inferior**	**Bone**	**Graft**	**IBN-like**	**Intra-soft**	**Extra-soft**	**Other**
Bio-Oss	15.5 ± 5.7	38.2 ± 11.6	0.0 ± 0.0	37.7 ± 8.1	0.0 ± 0.0	8.6 ± 2.0
SP-Block	18.9 ± 9.6	15.2 ± 11.0	0.0 ± 0.0	55.2 ± 15.7	0.0 ± 0.0	10.7 ± 2.6
ReproBone	14.4 ± 9.2	20.6 ± 3.7	16.6 ± 3.3	43.8 ± 3.3	0.0 ± 0.0	4.6 ± 3.0
Alos	17.2 ± 10.3	16.6 ± 5.6	0.0 ± 0.0	49.9 ± 11.3	0.2 ± 0.3	16.2 ± 3.3

## Data Availability

The original contributions presented in the study are included in the article; further inquiries can be directed to the corresponding author.

## References

[1] Araújo M.G., Sonohara M., Hayacibara R., Cardaropoli G., Lindhe J. (2002). Lateral ridge augmentation by the use of grafts comprised of autologous bone or a biomaterial. An experiment in the dog. J. Clin. Periodontol..

[2] Schropp L., Wenzel A., Kostopoulos L., Karring T. (2003). Bone healing and soft tissue contour changes following single-tooth extraction: A clinical and radiographic 12-month prospective study. Int. J. Periodontics Restor. Dent..

[3] Avila-Ortiz G., Elangovan S., Kramer K.W., Blanchette D., Dawson D.V. (2014). Effect of alveolar ridge preservation after tooth extraction: A systematic review and meta-analysis. J. Dent. Res..

[4] Cordaro L., Amadé D.S., Cordaro M. (2002). Clinical results of alveolar ridge augmentation with mandibular block bone grafts in partially edentulous patients prior to implant placement. Clin. Oral Implant. Res..

[5] Chiapasco M., Zaniboni M., Rimondini L. (2007). Autogenous onlay bone grafts vs. alveolar distraction osteogenesis for the correction of vertically deficient edentulous ridges: A 2–4-year prospective study on humans. Clin. Oral Implant. Res..

[6] Elboraey M.O., Alqutaibi A.Y., Aboalrejal A.N., Borzangy S., Zafar M.S., Al-Gabri R., Alghauli M.A., Ramalingam S. (2025). Regenerative approaches in alveolar bone augmentation for dental implant placement: Techniques, biomaterials, and clinical decision-making: A comprehensive review. J. Dent..

[7] Habal M.B. (1994). Bone grafting in craniofacial surgery. Clin. Plast. Surg..

[8] Marx R.E. (2007). Bone and bone graft healing. Oral Maxillofac. Surg. Clin. North Am..

[9] Louis P.J., Sittitavornwong S. (2019). Managing Bone Grafts for the Mandible. Oral Maxillofac. Surg. Clin. N. Am..

[10] Christensen J.G., Grønlund G.P., Georgi S.R., Starch-Jensen T., Bruun N.H., Jensen S.S. (2023). Horizontal Alveolar Ridge Augmentation with Xenogenic Block Grafts Compared with Autogenous Bone Block Grafts for Implant-retained Rehabilitation: A Systematic Review and Meta-Analysis. J. Oral Maxillofac. Res..

[11] Chana P., O’Malley L., Yates J. (2026). Non-autogenous vs. autogenous bone block grafts for alveolar ridge augmentation: A systematic review. Oral Surg..

[12] Donkiewicz P., Benz K., Kloss-Brandstätter A., Jackowski J. (2021). Survival Rates of Dental Implants in Autogenous and Allogeneic Bone Blocks: A Systematic Review. Medicina.

[13] Nkenke E., Neukam F.W. (2014). Autogenous bone harvesting and grafting in advanced jaw resorption: Morbidity, resorption and implant survival. Eur. J. Oral Implantol..

[14] Starch-Jensen T., Deluiz D., Deb S., Bruun N.H., Tinoco E.M.B. (2020). Harvesting of Autogenous Bone Graft from the Ascending Mandibular Ramus Compared with the Chin Region: A Systematic Review and Meta-Analysis Focusing on Complications and Donor Site Morbidity. J. Oral Maxillofac. Res..

[15] Guan D., Zhao R., Guo Y., Li J., Ma N., Gong J. (2023). Efficacy of autogenous tooth block for lateral ridge augmentation compared with autogenous bone block: A systematic review and meta-analysis. Medicine.

[16] Bozza B., Pesce P., Baldi D., Bagnasco F., Migliorati M., De Angelis N. (2025). Synthetic Biomaterials for Alveolar Bone Regeneration: A Systematic Review of Clinical Evidence. Materials.

[17] Wang H., Kang J. (2026). Bone grafts and synthetic substitutes in dental applications: A comprehensive review of molecular mechanisms, materials evolution, and clinical perspective. Front Bioeng. Biotechnol..

[18] Hnitecka S., Olchowy C., Olchowy A., Dąbrowski P., Dominiak M. (2024). Advancements in alveolar bone reconstruction: A systematic review of bone block utilization in dental practice. Dent. Med. Probl..

[19] Liu H.P., Chiam S.Y., Kakar E., Wang H.L. (2025). Xenogeneic Bone Block in Lateral Ridge Augmentation: Where Are We Now? A Systematic Review and Meta-analysis. Int. J. Oral Maxillofac. Implant..

[20] Tang Y., Zhai S., Yu H., Qiu L. (2023). Clinical feasibility evaluation of a digital workflow of prosthetically oriented onlay bone grafting for horizontal alveolar augmentation: A prospective pilot study. BMC Oral Health.

[21] De Santis E., Lang N.P., Favero G., Beolchini M., Morelli F., Botticelli D. (2015). Healing at mandibular block-grafted sites. An experimental study in dogs. Clin. Oral Implant. Res..

[22] Haugen H.J., Sanz J., Perale G., Saiz A.M., Romandini M., Rahmati M. (2026). Bone Grafts: Everything You Need to Know. J. Periodontal Res..

[23] Sakaguchi R., Xavier S.P., Morinaga K., Botticelli D., Silva E.R., Nakajima Y., Baba S. (2023). Histological Comparison of Collagenated Cancellous Equine Bone Blocks Used as Inlay or Onlay for Lateral Bone Augmentation in Rabbits. Materials.

[24] Kaneko N., Xavier S.P., Morinaga K., Botticelli D., Silva E.R., Nakajima Y., Baba S. (2023). Implants Placed with a Ring Technique Using Inlay and Onlay Block Xenografts in the Mandible of Rabbits. Materials.

[25] Baldini N., De Sanctis M., Ferrari M. (2011). Deproteinized bovine bone in periodontal and implant surgery. Dent. Mater..

[26] Figueiredo A., Coimbra P., Cabrita A., Guerra F., Figueiredo M. (2013). Comparison of a xenogeneic and an alloplastic material used in dental implants in terms of physico-chemical characteristics and in vivo inflammatory response. Mater. Sci. Eng. C Mater. Biol. Appl..

[27] Schmitt C.M., Doering H., Schmidt T., Lutz R., Neukam F.W., Schlegel K.A. (2013). Histological results after maxillary sinus augmentation with Straumann^®^ BoneCeramic, Bio-Oss^®^, Puros^®^, and autologous bone. A randomized controlled clinical trial. Clin. Oral Implant. Res..

[28] Piattelli M., Favero G.A., Scarano A., Orsini G., Piattelli A. (1999). Bone reactions to anorganic bovine bone (Bio-Oss) used in sinus augmentation procedures: A histologic long-term report of 20 cases in humans. Int. J. Oral Maxillofac. Implant..

[29] Valentini P., Abensur D., Wenz B., Peetz M., Schenk R. (2000). Sinus grafting with porous bone mineral (Bio-Oss) for implant placement: A 5-year study on 15 patients. Int. J. Periodontics Restor. Dent..

[30] Jensen S.S., Bosshardt D.D., Gruber R., Buser D. (2014). Long-term stability of contour augmentation in the esthetic zone: Histologic and histomorphometric evaluation of 12 human biopsies 14 to 80 months after augmentation. J. Periodontol..

[31] Canellas J.V.D.S., Soares B.N., Ritto F.G., Vettore M.V., Vidigal Júnior G.M., Fischer R.G., Medeiros P.J.D. (2021). What grafting materials produce greater alveolar ridge preservation after tooth extraction? A systematic review and network meta-analysis. J. Cranio-Maxillofac. Surg..

[32] Pistilli R., Felice P., Piatelli M., Nisii A., Barausse C., Esposito M. (2014). Blocks of autogenous bone versus xenografts for the rehabilitation of atrophic jaws with dental implants: Preliminary data from a pilot randomised controlled trial. Eur. J. Oral Implantol..

[33] Eliaz N., Metoki N. (2017). Calcium Phosphate Bioceramics: A Review of Their History, Structure, Properties, Coating Technologies and Biomedical Applications. Materials.

[34] Rezwan K., Chen Q.Z., Blaker J.J., Boccaccini A.R. (2006). Biodegradable and bioactive porous polymer/inorganic composite scaffolds for bone tissue engineering. Biomaterials.

[35] Félix Lanao R.P., Jonker A.M., Wolke J.G., Jansen J.A., van Hest J.C., Leeuwenburgh S.C. (2013). Physicochemical properties and applications of poly(lactic-co-glycolic acid) for use in bone regeneration. Tissue Eng. Part B Rev..

[36] Gentile P., Chiono V., Carmagnola I., Hatton P.V. (2014). An overview of poly(lactic-co-glycolic) acid (PLGA)-based biomaterials for bone tissue engineering. Int. J. Mol. Sci..

[37] Chai Y.C., Carlier A., Bolander J., Roberts S.J., Geris L., Schrooten J., Van Oosterwyck H., Luyten F.P. (2012). Current views on calcium phosphate osteogenicity and the translation into effective bone regeneration strategies. Acta Biomater..

[38] Toledano M., Asady S., Toledano-Osorio M., García-Godoy F., Serrera-Figallo M.A., Benítez-García J.A., Osorio R. (2020). Differential Biodegradation Kinetics of Collagen Membranes for Bone Regeneration. Polymers.

[39] Pinotti F.E., Pimentel Lopes de Oliveira G.J., Scardueli C.R., Costa de Medeiros M., Stavropoulos A., Chiérici Marcantonio R.A. (2018). Use of a Non-Crosslinked Collagen Membrane During Guided Bone Regeneration Does Not Interfere With the Bone Regenerative Capacity of the Periosteum. J. Oral Maxillofac. Surg..

[40] Papavasileiou D.A., Leventis M., Agrogiannis G., Kalyvas D. (2025). Effect of Isolating the Periosteum With a Resorbable Barrier Membrane on Neoangiogenesis in Guided Bone Regeneration: An Experimental Study. Cureus.

[41] Kondo T., Kanayama K., Egusa H., Nishimura I. (2023). Current perspectives of residual ridge resorption: Pathological activation of oral barrier osteoclasts. J. Prosthodont Res..

[42] Kotsu M., Apaza Alccayhuaman K.A., Ferri M., Iezzi G., Piattelli A., Fortich Mesa N., Botticelli D. (2022). Osseointegration at Implants Installed in Composite Bone: A Randomized Clinical Trial on Sinus Floor Elevation. J. Funct. Biomater..

[43] Tanaka K., Botticelli D., Canullo L., Baba S., Xavier S.P. (2020). New bone ingrowth into β-TCP/HA graft activated with argon plasma: A histomorphometric study on sinus lifting in rabbits. Int. J. Implant Dent..

[44] Yanagiuchi H., Botticelli D., Silva E.R., Xavier S.P., Iezzi G., Uwazumi T., Baba S. (2026). Comparative Histological and Histomorphometric Analysis of Xenografts and Synthetic Biomaterials in Rabbit Sinus Floor Elevation. J. Funct. Biomater..

[45] Silva E.R., Balan V.F., Botticelli D., Soldini C., Okamoto R., Xavier S.P. (2021). Histomorphometric, Immunohistochemical and Microtomographic Comparison between Autogenous and Xenogenous Bone Blocks for Mandibular Lateral Augmentation in Rabbits. Materials.

[46] Campillo V.E., Langonnet S., Pierrefeu A., Chaux-Bodard A.G. (2014). Anatomic and histological study of the rabbit mandible as an experimental model for wound healing and surgical therapies. Lab Anim..

[47] Pearce A.I., Richards R.G., Milz S., Schneider E., Pearce S.G. (2007). Animal models for implant biomaterial research in bone: A review. Eur. Cell Mater..

